# Prediction of adult asthma risk in early childhood using novel adult asthma predictive risk scores

**DOI:** 10.1111/all.15876

**Published:** 2023-09-03

**Authors:** Abdal J. Farhan, Dilini M. Kothalawala, Ramesh J. Kurukulaaratchy, Raquel Granell, Angela Simpson, Clare Murray, Adnan Custovic, Graham Roberts, Hongmei Zhang, S. Hasan Arshad

**Affiliations:** ^1^ The David Hide Asthma and Allergy Research Centre St. Mary's Hospital Isle of Wight UK; ^2^ Clinical and Experimental Sciences, Faculty of Medicine University of Southampton Southampton UK; ^3^ NIHR Biomedical Research Centre University Hospital Southampton Southampton UK; ^4^ Human Development and Health, Faculty of Medicine University of Southampton Southampton UK; ^5^ MRC Integrative Epidemiology Unit, Population Health Sciences, Bristol Medical School University of Bristol Bristol UK; ^6^ Division of Infection, Immunity and Respiratory Medicine, School of Biological Sciences The University of Manchester, Manchester Academic Health Science Centre, and Manchester University NHS Foundation Trust Manchester UK; ^7^ National Heart and Lung Institute Imperial College London London UK; ^8^ Division of Epidemiology, Biostatistics, and Environmental Health, School of Public Health University of Memphis Memphis Tennessee USA

**Keywords:** allergic sensitisation, asthma, prediction, risk scores, wheeze

## Abstract

**Background:**

Numerous risk scores have been developed to predict childhood asthma. However, they may not predict asthma beyond childhood. We aim to create childhood risk scores that predict development and persistence of asthma up to young adult life.

**Methods:**

The Isle of Wight Birth Cohort (*n* = 1456) was prospectively assessed up to 26 years of age. Asthma predictive scores were developed based on factors during the first 4 years, using logistic regression and tested for sensitivity, specificity and area under the curve (AUC) for prediction of asthma at (i) 18 and (ii) 26 years, and persistent asthma (PA) (iii) at 10 and 18 years, and (iv) at 10, 18 and 26 years. Models were internally and externally validated.

**Results:**

Four models were generated for prediction of each asthma outcome. ASthma PredIctive Risk scorE (ASPIRE)‐1: a 2‐factor model (recurrent wheeze [RW] and positive skin prick test [+SPT] at 4 years) for asthma at 18 years (sensitivity: 0.49, specificity: 0.80, AUC: 0.65). ASPIRE‐2: a 3‐factor model (RW, +SPT and maternal rhinitis) for asthma at 26 years (sensitivity: 0.60, specificity: 0.79, AUC: 0.73). ASPIRE‐3: a 3‐factor model (RW, +SPT and eczema at 4 years) for PA‐18 (sensitivity: 0.63, specificity: 0.87, AUC: 0.77). ASPIRE‐4: a 3‐factor model (RW, +SPT at 4 years and recurrent chest infection at 2 years) for PA‐26 (sensitivity: 0.68, specificity: 0.87, AUC: 0.80). ASPIRE‐1 and ASPIRE‐3 scores were replicated externally. Further assessments indicated that ASPIRE‐1 can be used in place of ASPIRE‐2‐4 with same predictive accuracy.

**Conclusion:**

ASPIRE predicts persistent asthma up to young adult life.

## Graphical abstract

We aimed to create childhood risk scores for adult asthma prediction. Four ASPIRE models were developed in the Isle of Wight cohort that has been followed to young adult life and validated internally and externally. In the high‐risk score category, the maximum risk of adult asthma ranged from 80 to 100%. The predictive accuracy was highest for persistent adult asthma.Abbreviations: ASPIRE, Adult Asthma Predictive Risk Scores; ROC, receiver operating characteristic

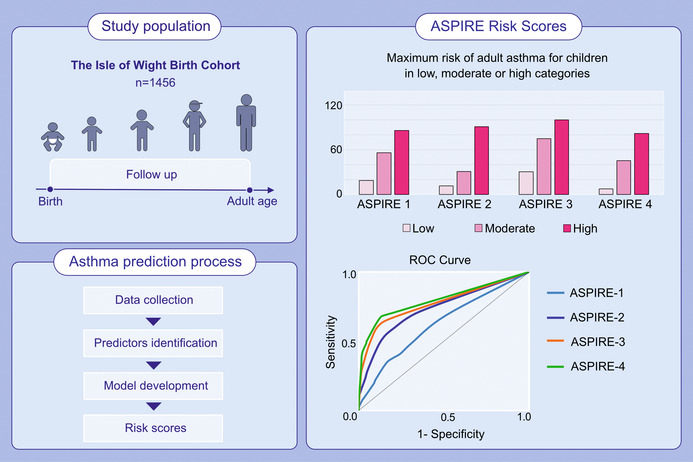

## 1 INTRODUCTION

Early childhood wheezing is common but its long‐term outcome in relation to asthma beyond childhood remains poorly defined. Risk scores have been developed in numerous birth cohorts to predict childhood asthma development^1^, ^2^, ^3^, ^4^, ^5^, ^6^, ^7^, ^8^ (recently reviewed^9^, ^10^). These approaches could identify children at high risk of asthma for preventive strategies, promote personalised care by targeted use of asthma medications and reduce the wastage of healthcare resources.^11^ While the ‘asthma predictive index’ was the first to gain widespread recognition for childhood asthma prediction,^1^ recently developed risk scores such as predictive asthma risk score (PARS) have shown improved predictive performance.^8^ However, the relevance of such scores to adult asthma status remains unknown.

The natural history of asthma is one of remission, relapse and new onset over the life course, and these events are particularly common during adolescence and early adult life.^12^, ^13^, ^14^, ^15^ Hence, risk scores developed for childhood asthma may not have the same relevance for later adult asthma or persistent asthma from childhood to adulthood. Asthma that persists from childhood to adulthood is associated with worse adult lung function and greater disease morbidity.^16^ Previous studies have reported association of early life risk factors for adult or adult‐onset asthma.^17^, ^18^, ^19^, ^20^ Balemans et al developed prediction rules based on risk factors identified at age 2 and 4 in a Dutch cohort followed up to age 21 but the power for both models was low.^21^ However, no previous study has attempted to develop scores that can be used to predict asthma that persists from childhood.

In this study, we developed predictive models within the Isle of Wight Birth Cohort (IOWBC). Based on these models, risk scores were created to predict (i) asthma diagnosed at age 18 (Asthma‐18), at age 26 (Asthma‐26), at both 10 and 18 years (PA‐18), and persistent asthma, that is asthma diagnosed at all 3 assessments of 10, 18 and 26 years (PA‐26). Models were replicated (up to age 18) in two independent cohorts: The Manchester Asthma and Allergy Study (MAAS) and The Avon Longitudinal Study of Parents and Children (ALSPAC). We also tested if the best existing childhood score (PARS) reported in the literature^8^ can be extended to predict adult asthma and compared our model with that of PARS in its ability to predict adult asthma.

## 2 METHODS

### 2.1 The Isle of Wight birth cohort (IOWBC)

A whole population birth cohort was established on the Isle of Wight. Parents of 1456 children (of 1536 born between January 1989 and February 1990) consented for the longitudinal study (95.8%). Ethical approval was obtained from the Isle of Wight NHS Ethic committee (No 05/89; dated 08/22/1988), at the time of recruitment and subsequently at each visit. Participants were seen at 1‐year (*n* = 1369; 94%), 2‐year (*n* = 1231; 84.5%), 4‐year (*n* = 1218; 83.7%), 10‐year (*n* = 1373; 94.3%), 18‐year (*n* = 1313; 90.1%) and 26‐year (*n* = 1033; 70.9%). Participants completed detailed assessments at each visit to ascertain asthma and allergy status and provide information on environmental exposures. Detailed methodology is provided in the on‐line supplement and has been reported previously.^22^

### 2.2 Definitions of variables

Recurrent wheeze (RW): defined as three or more separate episodes of wheeze occurring in the past 12 months at 4 years.

Allergic sensitisation: ‘positive SPT’ (at least 3 mm greater than negative control) to one or more allergens at age 4 years. Allergens included grass pollen mix, cat epithelia, dog epithelia, cladosporium herbarum, alternaria alternate, house dust mite, hen's egg, milk, soya, cod, wheat and peanut.

Current asthma: physician diagnosed asthma ever and either current wheeze or currently on asthma medication. Current asthma was evaluated at 10, 18 and 26 years. (see Table S1 for definitions and prevalences). Adult aSthma PredIctive Risk scorEs (ASPIRE) were developed for each of the four asthma outcomes:

1. ASPIRE‐1 for late adolescent asthma diagnosed at age 18 (Asthma‐18)
2. ASPIRE‐2 for young adult asthma diagnosed at age 26 (Asthma‐26)
3. ASPIRE‐3 for persistent asthma to late adolescence, that is asthma diagnosed at both ages of 10 and 18 years (PA‐18)
4. ASPIRE‐4 for persistent asthma diagnosis to young adulthood, that is asthma diagnosed at all three assessments of 10, 18 and 26 years (PA‐26)

We then applied our simplest model (ASPIRE‐1), initially developed for Asthma‐18 to Asthma‐26, PA‐18 and PA‐26, to assess whether this 2‐factor model can be applied universally without loss of predictability, as these two factors (RW and + SPT) were common to all four models. All models were replicated internally, and where possible, externally in two independent cohorts. As our asthma definitions were questionnaire based, we used spirometry carried out at 10, 18 and 26 years as alternative outcomes (on‐line Table S1).

### 2.3 Statistical analysis

All ASPIRE models were developed using relevant factors available within the first 4 years of life that had potential relevance for asthma outcomes based on the published literature (Tables S2 and S3). All factors were tested for their association with each of the four asthma outcomes using chi‐squared tests (Fisher's exact test if the frequency in 20% values in a cell were below 5). Statistically significant (defined as *p* < .05) factors in the univariate models for any asthma outcomes (Asthma‐18, Asthma‐26, PA‐18 and PA‐26) were included in separate logistic regression models for their respective asthma outcomes. A backward stepwise variable selection approach was used to select independent factors to develop the final model for each outcome. In particular, we start from the full model with all the variables in the model. At each step, one variable was excluded if the variable's *p*‐value was the largest and larger than .05. This process was continued until all the variables left in the model were statistically significant (<0.05). Variables with *p*‐value < .05 were included in the final model, and the odds ratio (OR) for each predictor was calculated. Factors obtained from the logistic regression model were internally validated using bootstrapping (see on‐line supplement). For each bootstrap sample, we fitted the same logistic regression model as that for the original data and stored the coefficients.

An individual risk score was calculated for each predictor by rounding the OR to the nearest integer. Consequently, for each model, a cumulative score was calculated for each subject by adding up the rounded ORs. Model precision was evaluated using the Hosmer–Lemeshow goodness‐of‐fit statistic. The calculated scores along with asthma status were then used to estimate the area under the curve (AUC) and its 95% confidence interval. A threshold of cumulative score was selected such that it maximised sensitivity and specificity, positive predictive value (PPV), negative predictive value (NPV) and likelihood ratios (LR), which were calculated for all models based on the selected threshold.

For a more practical risk estimation, we further classified the scores into three risk categories. A weight was assigned to each factor by rounding the OR to the nearest whole number. These weights were then summed to calculate a risk score for each subject. Overall, the scores range from 0 to 22 for all four models. For each model, the scores were divided into three categories using quartiles; scores in the first quartile were categorised as ‘low risk’, scores in the second and third quartiles were categorised as ‘moderate risk’, and score in the fourth quartile was categorised as ‘high risk’.^23^

As a validation, we looked at lung function as an outcome for ASPIRE‐1. Spirometry was carried out at ages 10 (*n* = 981), 18 (*n* = 839) and 26 years (*n* = 547), following American Thoracic Society (ATS) guidelines (see Appendix S1 for details).

### 2.4 Extending PARS to predict adult asthma status

PARS scores were calculated for each individual in the IOWBC. The PARS scores were obtained using 5 out of the 6 factors included in the original PARS model: parental asthma, eczema before 4 years, wheezing apart from colds, early wheezing (before 4 years) and positive SPT (+SPT) to 2 or more allergens. ‘African American race’, used in the original PARS score, was excluded in this analysis as it was not relevant to the predominantly (98%) Caucasian IOWBC population. AUC was calculated to assess the PARS scores for the prediction of Asthma‐18, Asthma‐26, PA‐18 and PA‐26.

### 2.5 External validation

External validation was performed to assess the generalisability of the developed models in two independent population‐based cohorts: (i) MAAS and (ii) ALSPAC.

MAAS (*n* = 1211) is an unselected birth cohort based in Manchester, UK. Children were followed up from birth to age 18 years. ALSPAC is an unselected birth cohort based in Bristol, UK. It recruited more than 14,000 pregnant women; children arising from the pregnancy have been followed up for more than two decades. Details are provided in the on‐line supplement. Factors considered in the replication analyses were selected to match those in IOWBC as close as possible (Table S1). To replicate ASPIRE models in MAAS and ALSPAC, we used the same weights (rounded regression coefficients) derived in the IOWBC to calculate risk scores for each individual in MAAS and ALSPAC.

All statistical analyses were performed in SPSS (V 25.0).

## 3 RESULTS

Of the total cohort of 1456 Participants, 905 (62%) participants were included in ASPIRE‐1, 718 (49%) in ASPIRE‐2, 677 (46%) in ASPIRE‐3 and 557 (38%) in ASPIRE‐4. Tables S4 and S5 show that the population included in various analyses were similar to the whole cohort, minimising selection bias. Table S1 provides prevalence of asthma and early childhood factors used to develop ASPIRE models.

In the univariate analysis, we included 31 potential factors and exposures occurring during the first 4 years to identify statistically significant risk factors at univariate level for each asthma outcome (Tables S2 and S3).

### 3.1 ASPIRE‐1: Prediction of Asthma‐18

**FIGURE 1:**
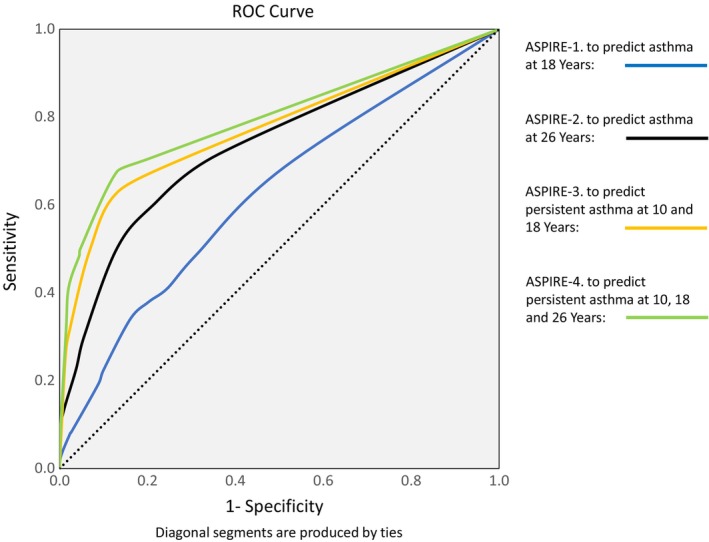
Sensitivity, specificity and area under the curve for different models. Receiver operating characteristic (ROC) curves comparing 4 ASPIRE models. Model discrimination was evaluated by the area under the ROC curve. Model discrimination was excellent for persistent asthma between 10 and 18 years (AUC = 0.77) and 10, 18 and 26 (AUC = 0.80), and both were better than asthma at age 18 (AUC = 0.65) and at age 26 (AUC = 0.73).

Twelve factors were identified for Asthma‐18 in the univariate model (Table S2A). These were included in multivariable logistic regression analysis, where we identified RW and + SPT at 4 years as statistically significant factors (Table 1), which were used to calculate sensitivity (0.49), specificity (0.80) and AUC (0.65, 95% CI: 0.61–0.70) (Table 2, Figure 1). Risk of asthma at 18 years was further classified as low for scores (0), moderate (3) and high (6) (Table 3). Young children with both risk factors had a 6‐fold higher odds of asthma at 18 years compared to children without this risk.

**TABLE 1:** All models factors, scores and bootstrapping validation.

Model	Factors	*p*‐Value	Adjusted OR	95% CI		Scores
				Lower	Upper	
ASPIRE‐1 for Asthma‐18						
ASPIRE Model	RW at 4 years	<.001	3.42	2.22	5.25	3
	+SPT at 4 years	<.001	2.97	1.99	4.45	3
Bootstrapping	RW at 4 years	<.001	3.06	2.11	4.47	
	+SPT at 4 years	<.001	2.99	2.05	4.36	
ASPIRE‐2 for Asthma‐26						
ASPIRE Model	RW at 4 years	<.001	4.72	2.75	8.09	5
	+SPT at 4 years	<.001	3.03	1.78	5.13	3
	Maternal rhinitis	.001	2.42	1.43	4.08	2
Bootstrapping	RW at 4 years	.001	4.54	2.81	7.61	
	+SPT at 4 years	.001	2.89	1.78	4.92	
	Maternal rhinitis	.001	2.42	1.49	3.96	
ASPIRE‐3 for PA‐18						
ASPIRE Model	RW at 4 years	<.001	14.11	7.46	26.67	14
	+SPT at 4 years	<.001	4.23	2.43	7.36	4
	Eczema at 4 years	.034	2.04	1.05	3.93	2
Bootstrapping	RW at 4 years	<.001	11.81	6.78	22.07	
	+SPT at 4 years	<.001	3.99	2.38	7.12	
	Eczema age at 4 years	.001	2.50	1.42	4.54	
ASPIRE‐4 for PA‐26						
ASPIRE Model	RW at 4 years	<.001	11.94	5.62	25.38	12
	+SPT at 4 years	<.001	7.02	3.52	13.99	7
	Recurrent chest infections at 2 years	.049	2.52	1.00	6.30	3
Bootstrapping	RW at 4 years	.001	10.70	5.25	22.30	
	+SPT at 4 years	.001	5.66	2.80	12.64	
	Recurrent chest infections at 2 years	.035	2.25	0.98	4.39	

*Note*: All factors were tested for their association with each of the four asthma outcomes using chi‐squared tests. Statistically significant (defined as *p* < .05) factors in the univariate models for any asthma outcomes (Asthma‐18, Asthma‐26, PA‐18 and PA‐26) were included in separate logistic regression models for their respective asthma outcomes. A backward stepwise variable selection approach was used to select independent factors to develop the final model for each outcome. Factors with *p*‐value <.05 were included in the final model, and the odds ratio (OR) for each predictor was calculated. Factors obtained from the logistic regression model were internally validated using bootstrapping. For each bootstrap sample, we fitted the same logistic regression model as that for the original data and stored the coefficients.

Abbreviations: +SPT, positive skin prick test; ASPIRE, Adult aSthma PredIctive Risk score (adjusted odds ratios); CI, confidence intervals; RW, recurrent wheeze.

**TABLE 2:** Performance of ASPIRE‐1 to ASPIRE‐4 models in IOWBC and replication cohorts (MAAS and ALSPAC).

Model	Participants		AUC 95% CI	Sensitivity	Specificity	PPV	NPV	Accuracy	LR+	LR‐
	Included %	Total								
ASPIRE‐1 for Asthma‐18 (wheeze, SPT)	905 (62%)	1456	0.65 0.61–070	0.49	0.80	0.47	0.81	0.72	2.44	0.64
ASPIRE‐1 replicated in MAAS	548 (47%)	1163	0.69 0.64–0.74	0.58	0.77	0.59	0.76	0.70	2.55	0.55
ASPIRE‐1 replicated in ALSPAC	4370 (31%)	14,152	0.64 0.61–0.66	0.45	0.82	0.25	0.92	0.77	2.46	0.67
ASPIRE‐2 for Asthma‐26 (wheeze, SPT, maternal rhinitis)	718 (49%)	1456	0.73 0.67–0.79	0.60	0.79	0.32	0.92	0.76	2.83	0.51
ASPIRE‐3 for PA‐18 (wheeze, SPT, eczema)	677 (46%)	1456	0.77 0.72–0.82	0.63	0.87	0.57	0.89	0.81	4.70	0.42
ASPIRE‐3 replicated in MAAS	439 (38%)	1163	0.80 0.74–0.85	0.75	0.79	0.55	0.91	0.78	3.60	0.31
ASPIRE‐3 replicated in ALSPAC	3559 (25%)	14,152	0.71 0.68–0.75	0.59	0.83	0.23	0.96	0.81	3.50	0.49
ASPIRE‐4 for PA‐26 (wheeze, SPT, recurrent chest infection)	557 (38%)	1456	0.80 0.75–0.87	0.68	0.87	0.42	0.95	0.85	5.17	0.37

*Note*: Total scores (ORs) from each of the model were computed, and those scores were used to run the ROC analysis. AUC, sensitivity, specificity, PPV, NPV, accuracy, LR+ and LR‐ were obtained from the ROC analysis.

Abbreviations: ALSPAC, The Avon Longitudinal Study of Parents and Children; ASPIRE, Adult aSthma PredIctive Risk score; AUC, Area under the curve; IOWBC, Isle of Wight Birth Cohort; LR, Likelihood ratio; MAAS, The Manchester Asthma and Allergy Study; NPV, Negative predictive value; PPV, Positive predictive value; SPT, Skin Prick Test.

**TABLE 3:** ASPIRE Score risk levels for asthma at age 18 and 26 years and persistent (at 10, 18 and 26 years).

Scores	ASPIRE‐1. Risk of Asthma‐18	Interpretation
0	18.8%	Low risk
3	55.8%	Moderate risk
6	85.9%	High risk
ASPIRE‐2. Risk of Asthma‐26		
0	6.9%	Low risk
2	11.4%	
3	16.7%	Moderate risk
5	30.8%	
7	40%	High risk
8	37.1%	
10	90.9%	
ASPIRE‐3. Risk of PA‐18		
0	10.3%	Low risk
2	15.6%	
4	30.4%	
6	62.5%	Moderate risk
14	60.0%	
16	75.0%	
18	75.5%	High risk
20	100.0%	
ASPIRE‐4. Risk of PA‐26		
0	4.1%	Low risk
3	7.4%	
7	20.5%	Moderate risk
10	26.7%	
12	38.1%	
15	45.5%	
19	72.2%	High risk
22	81.8%	

*Note*: An individual risk score was calculated for each predictor by rounding the OR to the nearest integer. Children were categorised based on their risk of asthma estimated by their cumulative score, which translates into the proportion of children who will develop asthma (middle column), although the proportion in each category might differ between different models. To enhance ASPIRE's practical use in the clinics, we further classified scores into three risk categories using quartiles; ‘low, medium and high risk’.

The green colour indicates low risk, yellow indicates medium risk and red indicates high risk.

Abbreviation: ASPIRE, Adult aSthma PredIctive Risk score.

### 3.2 ASPIRE‐2: Prediction of Asthma‐26

Ten factors were identified for Asthma‐26 in the univariate model (Table S2B) to include in the multivariable logistic regression analysis, where we identified RW, +SPT and maternal rhinitis as independently significant (Table 1) These were used to calculate sensitivity (0.60), specificity (0.79) and AUC (0.73, 95% CI: 0.67–0.79) (Table 2, Figure 1). The risk of developing asthma at 26 years was further classified into low (0–2), moderate (3–5) and high (7–10) risk groups (Table 3). Young children with all three factors had 10‐fold higher odds of asthma at 26 years.

### 3.3 ASPIRE‐3: Prediction of persistent asthma to late adolescence (PA‐18)

Nineteen factors were statistically significant for PA‐18 in the univariate model (Table S3A), which were included in the multivariable logistic regression analysis, where we identified RW, +SPT and eczema as independently significant (Table 1). These were used to calculate sensitivity (0.63), specificity (0.87) and AUC (0.77, 95% CI: 0.72–0.82) (Table 2, Figure 1). The risk of developing PA‐18 was further classified into low risk for scores (0–4), moderate (6–16) and high (18–20) (Table 3). Young children with all three factors had a 20‐fold higher odds of PA‐18 (Table 3).

### 3.4 ASPIRE‐4: Prediction of persistent asthma to adulthood (PA‐26)

Thirteen factors were statistically significant for PA‐26 in the univariate model, which were included in the multivariable logistic regression analysis, where we identified RW, +SPT at 4 years and recurrent chest infection at 2 years as independently significant (Table 1). These were used to calculate sensitivity (0.68), specificity (0.87) and AUC (0.80, 95% CI: 0.75–0.87) (Table 2, Figure 1). The risk of PA‐26 was further classified into low risk for scores (0–3), moderate (6–16) and high (20–22) (Table 3). Young children with all three factors had a 22‐fold higher odds of PA‐26 (Table 3).

### 3.5 Internal validation

The significant factors obtained from the logistic models were internally validated using bootstrapping for all ASPIRE models (Table 1). These showed that OR of all factors obtained from the original model was well within the 95% CI of the bootstrapping model.

### 3.6 ASPIRE‐1: One ASPIRE model for all four asthma outcomes

To assess the possibility of using one model to predict all asthma outcomes, we evaluated each of the four models, on their ability to predict each of the four asthma outcomes. We found that prediction based on two factor model used for ASPIRE‐1 (RW and + SPT) can be applied to predict adolescent and adult asthma outcomes with no loss of quality (Table 4) indicating that the third factor identified for ASPIRE‐2‐4 in logistic regression models contributes little additional predictability.

**TABLE 4:** ASPIRE‐2, ASPIRE‐3 and ASPIRE‐4 retested using ASPIRE‐1 two factors (RW and + SPT).

Model	Participants		AUC 95% CI	Sensitivity	Specificity	PPV	NPV	Accuracy	LR+	LR‐
	Included %	Total								
ASPIRE‐2 for Asthma‐26	718 (49%)	1456	0.73 0.67–0.79	0.60	0.79	0.32	0.92	0.76	2.83	0.51
ASPIRE‐1 for Asthma‐26	722 (49%)	1456	0.71 0.65–0.77	0.60	0.79	0.32	0.92	0.76	2.84	0.50
ASPIRE‐3 for PA‐18	677 (46%)	1456	0.77 0.72–0.82	0.63	0.87	0.57	0.89	0.81	4.70	0.42
ASPIRE‐1 for PA‐18	677 (46%)	1456	0.76 0.71–0.81	0.63	0.87	0.57	0.89	0.81	4.70	0.42
ASPIRE‐4 for PA‐26	557 (38%)	1456	0.80 0.75–0.87	0.68	0.87	0.42	0.95	0.85	5.17	0.37
ASPIRE‐1 for PA‐26	612 (42%)	1456	0.80 0.72–0.86	0.68	0.86	0.40	0.95	0.84	5.03	0.37

*Note*: Total scores (ORs) from each of the model were computed, and those scores were used to run the ROC analysis. AUC, sensitivity, specificity, PPV, NPV, accuracy, LR+ and LR‐ were obtained from the ROC analysis.

Abbreviations: ASPIRE, Adult aSthma PredIctive Risk score; AUC, Area under the curve; NPV, Negative predictive value; PA‐18, Persistent asthma at ages 10 and 18; PA‐26, persistent asthma at ages 10, 18 and 26; PPV, Positive predictive value; LR, Likelihood ratio; RW, recurrent wheeze; SPT, Skin Prick Test.

Analysis of lung function as an outcome at age 10, 18 and 26 years found that children with RW and + SPT had a higher risk of airway obstruction on pre‐bronchodilator spirometry at ages 18 and 26 years (Table S6).

### 3.7 Evaluating PARS to predict Asthma at 18 and 26 years and persistent asthma

We applied PARS to predict Asthma‐18, Asthma‐26, PA‐18 and PA‐26 and found it to have lower predictability compared to ASPIRE (Table 5). The predictive performance of PARS for adolescent and adult asthma was also inferior to its childhood value (AUC: 0.65–0.73 for adolescent and adult asthma, compared to 0.8 for asthma at age 10).^8^

**TABLE 5:** Comparison of ASPIRE with an existing childhood prediction model PARS at age 10/11 years and extended to predict asthma up to 26 years.

Model	Participants		AUC 95%CI	Sensitivity	Specificity	PPV	NPV	Accuracy	LR+	LR‐
	Included %	Total								
ASPIRE‐1 to predict Asthma‐18	905 (62%)	1456	0.65 0.61–070	0.49	0.80	0.47	0.81	0.72	2.44	0.64
PARS to predict Asthma‐18	1273 (87%)	1456	0.62 0.59–0.66	0.48	0.69	0.37	0.78	0.64	1.57	0.75
ASPIRE‐2 to predict Asthma‐26	718 (49%)	1456	0.73 0.67–0.79	0.60	0.79	0.32	0.92	0.76	2.83	0.51
PARS to predict Asthma‐26	998 (69%)	1456	0.62 0.57–0.67	0.51	0.68	0.22	0.88	0.65	1.58	0.72
ASPIRE‐3 to predict PA‐18	677 (46%)	1456	0.77 0.72–0.82	0.63	0.87	0.57	0.89	0.81	4.70	0.42
PARS to predict PA‐18	937 (64%)	1456	0.73 0.69–0.77	0.61	0.74	0.40	0.87	0.71	2.38	0.52
ASPIRE‐4 to predict PA‐26	557 (38%)	1456	0.80 0.75–0.87	0.68	0.87	0.42	0.95	0.85	5.17	0.37
PARS to predict PA‐26	857 (59%)	1456	0.71 0.65–0.77	0.61	0.75	0.22	0.94	0.73	2.39	0.53

*Note*: Total scores (ORs) from each of the model were computed, and those scores were used to run the ROC analysis. AUC, sensitivity, specificity, PPV, NPV, accuracy, LR+ and LR‐ were obtained from the ROC analysis.

Abbreviations: ASPIRE, Adult aSthma PredIctive Risk score; Asthma‐18, Asthma at 18 years; Asthma‐26, asthma at 26 years; AUC, Area under the curve; LR, Likelihood ratio; NPV, Negative predictive value; PA‐18, Persistent asthma at 18 years; PA‐26, Persistent asthma at 26 years; PARS, Predictive Asthma Risk scores; PPV, Positive predictive value.

### 3.8 Replicating ASPIRE in MAAS cohort

Of 1163 Participants, 548 (50%) and 539 (46%) were included in ASPIRE‐1 and ASPIRE‐3, respectively, for replication (Table 2), as data were not available in MAAS for age 26. Table S7 shows that the analysis population where data were available on relevant outcomes and factors was similar to the whole cohort. ASPIRE‐1 demonstrated good generalisability, offering slightly better performance to predict Asthma‐18; IOWBC vs MAAS (sensitivity: 0.49 vs. 0.58, specificity: 0.80 vs. 0.77 and AUC: 0.65 vs. 0.69) and ASPIRE‐3 (IOWBC vs MAAS: sensitivity: 0.63 vs. 0.75, specificity: 0.87 vs. 0.79 and AUC: 0.77 vs. 0.80).

### 3.9 Replicating ASPIRE in ALSPAC cohort

Of 14,152 participants, 4370 (31%) were included in replicating ASPIRE‐1 and 3642 (26%) in replicating ASPIRE‐3 (Table 2). The analysis population was similar to the whole cohort in terms of included factors (Table S8). Both ASPIRE‐1, IOWBC vs ALSPAC (sensitivity: 0.49 vs. 0.45, specificity: 0.80 vs. 0.82 and AUC: 0.65 vs. 0.64), and ASPIRE‐3, IOWBC vs ALSPAC (sensitivity: 0.63 vs. 0.55, specificity: 0.87 vs. 0.91 and AUC: 0.77 vs. 0.76), replicated well.

## 4 DISCUSSION

We identified early childhood factors for asthma at 18 and 26 years to construct a set of risk scores (ASPIRE‐1‐4) that can predict asthma in adolescence and young adulthood (Table 2). Interestingly, ASPIRE performed better for persistent asthma than for current asthma (Table 1). To our knowledge, this is the first report of development of risk scores to predict adolescent and young adult asthma, and importantly, asthma that persists until adulthood from information available during early childhood. An advantage of ASPIRE is that it is based on a few clinically relevant factors that are easily available to physicians. ASPIRE scores were developed in an unselected, population‐based cohort (IOWBC), using all available information and successfully validated internally, and ASPIRE‐1 and ASPIRE‐3 were externally replicated in two independent population‐based cohorts (MAAS and ALSPAC). Current best model to predict childhood asthma (PARS) could not be extended to effectively predict adolescent and adult asthma. We also found that ASPIRE‐1, a two factor model, can be used to predict adolescent and adult persistent asthma without loss of quality.

The natural history of asthma is one of relapse and remissions.^12^ Persistent asthma causes significantly higher morbidity and likelihood of complications.^16^, ^24^ Adolescence is a period of change, and we and others have shown significant changes in asthma status including remission of childhood asthma (often in boys) and new onset of asthma (often in girls) leading to the phenomenon of sex reversal.^15^, ^25^, ^26^, ^27^ Further, changes continue in the natural history of asthma beyond adolescence.^12^ PARS developed in the Cincinnati Childhood Allergy and Air Pollution Study and replicated in the IOWBC is currently the best model for childhood asthma prediction.^8^ However, we show that it has reduced ability to predict asthma beyond childhood and therefore cannot be repurposed to predict adult asthma. ASPIRE was superior to PARS for all adolescent/adult asthma outcomes (Table 5). We recommend using ASPIRE when assessing the risk of long‐term asthma morbidity.

We developed ASPIRE using a standard methodology, that is including all available risk factors and exposures in the first 4 years of life to identify those factors which are independently associated with our pre‐defined asthma outcomes which included point prevalence of asthma at age 18 and 26 and persistent asthma to late adolescence (age 18) and young adulthood (age 26). For Asthma‐18, we identified two factors (ASPIRE‐1) while for the other asthma outcomes, we identified three factors for each model (ASPIRE −2, −3 and − 4). RW and + SPT (ASPIRE‐1) were common factors across all ASPIRE models and predicted asthma outcomes with no loss of quality (Table 4). We would have expected to see improved predictability by the addition of a third factor given that the variable selection procedures identified the third factor in models 2–4 in the same way as the first two. However, the third factor had significantly lower OR than atopy and RW, indicating that most of the predictability was captured by atopy and RW. We propose that ASPIRE‐1 can be used to estimate the risk of adolescent/adult and persistent asthma. However, using ASPIRE‐2, ASPIRE‐3 and ASPIRE‐4 models for respective asthma outcomes provide a sophisticated grading of risk (Table 3), that is not available when using only two factors ASPIRE‐1 model.

We have previously proposed four factors to predict childhood asthma in the IOWBC,^2^ while PARS utilised six factors.^8^ There is variation in the numbers of predictors utilised in other studies to predict childhood asthma.^9^, ^10^ However, all share some combination of airway narrowing (wheezing/reduced lung function) with or without cold/viral infections, genetic predisposition (parental allergic diseases and/or genes) and a measure of atopy (+SPT, high IgE or eczema).^9^ These factors continue to be important beyond childhood and offer good predictive ability. In our study, susceptibility to airway narrowing was captured by RW, viral infections by recurrent chest infections, childhood atopy by +SPT and genetic predisposition by eczema and maternal rhinitis. Parental asthma did not reach significance in the multivariable analysis presumably because of its collinearity with other factors in the model such as atopy. The significance of atopy in the development of asthma has been consistently reported in multiple birth cohorts.^28^ However, its utilisation in asthma predictive algorithms has lagged behind. Our previous studies indicate that only 3–4 allergens need to be tested to define atopy with >95% confidence which further simplifies the implementation of ASPIRE in clinical practice.^29^, ^30^ Childhood wheeze is multifactorial and it is often associated with viral infections, which alone increases the risk of asthma.^31^ However, when combined with atopic predisposition, it increases the risk several folds and this risk profile (ASPIRE‐1) can be utilised to accurately predicts asthma that persists into adult life.

Prediction models have been developed in high‐risk populations using symptomatic preschool children with eczema and/or wheeze or they can be developed in a general population of pre‐schoolers.^9^ The latter has the benefit of not having to do pre‐screening before applying the predictive tools which increase their clinical utility. For our previous work on development of childhood predictive models, including PARS,^2^, ^8^ we have utilised unselected population. We therefore used the same strategy for ASPIRE, and with its 2 or 3 factors, it is easy to be applied in clinical practice for determination of risk for future asthma. It can be argued that including 10‐year data in our predictive model might have improved the accuracy further. However, given early origins of asthma,^32^ it is critical that young children are identified who are at risk of developing asthma that persists beyond childhood and carries higher morbidity.

Our data further support the importance of assessing allergic sensitisation in the management of preschool wheezers and should always be requested. A negative test would be highly reassuring, given high negative predictive values (Table 2). Similarly, a positive test indicates long‐term implications with increased risk of persistent asthma beyond childhood and deserves management considerations. Using ASPIRE 2–4 could be used to risk‐stratify participants for allergen avoidance measures based on specific sensitisation and early treatment with inhaled steroids possibly in those with higher risk stratification. For asthma prevention strategies, ASPIRE‐1 (RW and SPT) can be used as a surrogate outcome for persistent asthma and hence reduce the length of follow‐up required which makes these studies prohibitively expensive.

Our study has several strengths. First, each model was developed using logistic regression models to identify a combination of most relevant and independent factors to predict asthma within a general population, and thus applicable to paediatric patient population without any pre‐screening. Second, all models constructed to predict asthma were internally validated, while ASPIRE‐1 and ASPIRE‐3 were externally validated using two independent cohorts. Third, only few factors provide good predictability, and this information should be readily available to paediatricians and allergists. Fourth, the risk score is easy to calculate for each ASPIRE model (Table 3). Importantly, ASPIRE‐1 with 2 factors can be used to predict persistent adolescent and adult asthma outcomes with relatively high sensitivity and specificity. We show that children with RW and + SPT (ASPIRE‐1) have significant airway obstruction in later life. Indeed, it was interesting to note that they had worse lung function at ages 18 and 26 years than at age 10 years, which underscore the importance of early life factors for long‐term morbidity and it is consistent with the observation that ASPIRE performed better for persistent asthma. Finally, all ASPIRE models have high negative predictive value (Table 2), which could have utility in reassuring families with parental or sibling asthma or other risk factors without RW plus +SPT (Tables 2 and 4).

Our study has some limitations. Asthma diagnosis was based on questionnaires. However, this definition (physician diagnosed asthma plus symptoms and/or treatment) has been used extensively by us and others.^8^, ^13^, ^33^ The prediction for asthma at age 18 was modest (AUC: 0.65) but still those with high score had a 6‐fold higher asthma risk (Table 3). ASPIRE has relatively low sensitivity and PPV, and it was better at ruling out than ruling in of the disease. However, this may be useful if management decisions are based on the test. It might be useful to assess whether ASPIRE can detect long‐term asthma development at an age, earlier than 4 years. Unfortunately, we did not have skin prick test data in all children at our 1 and 2 years assessments. This is something to be considered in future cohorts. One limitation is that the population of both discovery cohort (IOWBC) and replications cohorts (MAAS and ALSPAC) are largely Caucasian, and therefore, ASPIRE requires replication in other ethnic groups. However, given the importance of viral infection and atopy in the development and persistence of asthma, it is likely that ASPIRE will be applicable to diverse populations.^28^ We used backward stepwise variable selection approach to choose variables for risk score calculation. Advanced approaches such as those with LASSO or adaptive LASSO penalty can be applied to eliminate variables with insignificant effect sizes although statistically significant.

In summary, we found that adolescent and adult persistent asthma can be predicted early in life using two or three factors that can be collected routinely at clinic visits with relatively high sensitivity, specificity and AUC. We suggest using ASPIRE‐1 to assess the risk of asthma that persists into adolescent and young adult with relatively high predictability and ASPIRE 3 and 4 if a graded risk assessment is needed. A major advantage of ASPIRE‐1 is its simplicity and applicability to most office/outpatient based practices where facility for skin prick test is available. Ability to predict asthma beyond childhood will facilitate early treatment to prevent avoidable morbidity.

## AUTHOR CONTRIBUTIONS

HA conceptualised the study. AJF performed data analysis, guided by HZ. AJF, RK, HZ and HA wrote the first draft. The text was then submitted to DMK, RG, AS, CM, AC and GR for review and editing. The text was revised, and further analysis was done where needed, in line with their comments. All authors have read and agreed to the final submitted draft.

## CONFLICT OF INTEREST STATEMENT

The authors declare no conflict of interests relevant to this work.

## Supporting information


Appendix S1.

## Data Availability

The data that support the findings of this study are available on request from the corresponding author. The data are not publicly available due to privacy or ethical restrictions.
