# Supplementary material for: Prediction of adult asthma risk in early childhood using novel adult asthma predictive risk scores
Source: Allergy. 2023 Sep 3;78(11):2969–79. doi: 10.1111/all.15876 (PMC10840748; doi:10.1111/all.15876)
Supplement: Supplementary file 1 — Appendix S1. [file ALL-78-2969-s001.docx]

**Supplementary Material**

**Title: Prediction of Adult Asthma-risk in early childhood using novel adult asthma predictive risk scores**

**Methods**

**Isle of Wight Birth Cohort**

Of the 1536 newborns born between January 1989 and February 1990, 1456 were recruited and prospectively followed up at 1, 2, 4, 10, 18 and 26-years of age.^1^ Ethical approval for the study was obtained from the local Research Ethics Committees at all assessments and informed consent obtained from the parents or participants as appropriate. At each visit, detailed questionnaires were completed by parents and/or participants seeking information on asthma and other allergic diseases as well as lifestyle and environmental exposures. Hospital records were mined to collect additional information not available through questionnaires and also to verify that provided by the participants. Physical examinations were performed in children with allergy-related symptoms at the 1-year and 2-year follow-ups, and in all participants who attended the Centre from the 4-year follow-up onwards. Skin prick testing (SPT) was performed at 4-years; (n=978; 67.7%), 10-years (n= 1036; 71.1%), 18-years (n= 851; 58.4%) and 26-years (n= 556; 38.2%) to a panel of common inhaled and food allergens (Biodiagnostics, Reinbek, Germany). A positive reaction was defined by a mean wheal diameter of ≥ 3 mm.^2^ Table S2 and S3 describe characteristics of participants included and analysed for each model.

**Manchester Asthma and Allergy Study (MAAS)**

The MAAS cohort is a longitudinal, whole-population birth cohort, established in 1995. 1211 women within the maternity catchment area of the Wythenshawe and Stepping Hill Hospitals were recruited into the study between 1995-1997. 1163 children were followed up for asthma and other atopic disorders at 1, 3, 5, 8, 11, 13-16 and 18-years.^3^ Ethical approval for the study was obtained from the local Research Ethics Committees at all assessments and informed consent obtained from the parents or participants as appropriate. Information was collected using validated questionnaires to collect data on allergy and asthma related symptoms and environmental exposures. Medical records were reviewed for further information. Blood samples were taken and lung function tests were performed from the three-year follow-up onwards. SPTs were performed from the 3-year follow-up against house dust mite (Dermatophagoides pteronyssinus), cat, dog, grass pollen, moulds, milk, and egg allergens. Factors considered in the replication analyses were selected to match those in IOWBC as close as possible (Table S1).

**The Avon Longitudinal Study of Parents and Children (ALSPAC),**

The Avon Longitudinal Study of Parents and Children (ALSPAC) is based at the University of Bristol, UK, also Known as Children of the 90s.^4,5^ The study recruited 15,454 pregnant women between April 1991 and December 1992; children arising from the pregnancy, and their partners were followed up for more than two decades at multiple timepoints. Ethical approval for the study was obtained from the ALSPAC Ethics and Law Committee and the Local Research Ethics Committees. Informed consent for the use of data collected via questionnaires and clinics was obtained from participants following the recommendations of the ALSPAC Ethics and Law Committee at the time. Information was collected using validated questionnaires on a wide spectrum of development and health matters including on allergy and asthma related symptoms and environmental exposures. Medical records were reviewed for further information. Blood samples were taken and skin tests were performed using standardised methodology to grass pollens, D. pteronyssinus, D. farinae, mixed tree pollen, cat, dog, horse, mouse, rabbit, guinea-pig, hamster, cockroach, alternaria alternata, cladosporium herbarium, aspergillus fumigatus, egg, milk, soya, cod, sesame seed, peanut. ALSPAC data are available through “data dictionary and variable search tool” at the following webpage: <http://www.bristol.ac.uk/alspac/researchers/our-data/>

Factors considered in the replication were selected to be as comparable as possible with those in the IOWBC (Table S1).

**Lung function assessment**

Pre-bronchodilator (pre-BD) spirometry was carried out at ages 10 (n=981), 18 (n=839) and 26-years (n=547) and post-bronchodilator (post-BD) spirometry at 18 (n=791) and 26-years (535). The subgroup with lung function data were not different in basic characteristics such as sex, smoking and allergic history to all the cohort members.^6^ For spirometry, American Thoracic Society (ATS) guidelines were followed to ensure validity and reproducibility. ^7^ Koko Spirometers (Longmont, CO, USA; <http://www.nspirehealth.com/products/koko-testing-devices/koko-sx-1000-spirometer/>) with calibration performed at least once daily were used. To perform spirometry, participants had to be free from respiratory infection for 14 days, not taking oral steroids, not taken beta_2_ agonist for 6 hours and abstained from caffeine intake for at least 4 hours. Spirometry was performed with participants standing without nose-clip. The acceptability criteria for each effort included a satisfactory start and end of test as well as a plateau in the volume–time curve. ^7^ FEV_1_, forced vital capacity (FVC), FEV_1_/FVC ratio, forced expiratory volume between 25 and 75 percent flow (FEF_25-75_) were recorded. As recommended, the highest of three FEV_1_ measurements within 5% of each other was used. Changes in FEV_1_, FEV_1_/FVC ratio, and FEF_25-75_ between assessments were calculated as the difference between values at 10 and 18 and then 18 and 26-years. Percent predicted for age, height, sex and ethnic origin was calculated for the FEV_1_, FVC, FEV_1_/FVC, and FEF_25-75_ based on Global Lung Initiative (GLI) reference equations.^8^

**Statistical analysis (Bootstrapping)**

To internally validate our findings, we used bootstrapping as the resampling strategy. To accomplish this goal, bootstrapping treats the data that we collected as one of many random samples that we could have collected and assigns measures of accuracy to sample estimates. It treats the original data as a representation of the whole population and resamples data repeatedly and randomly via drawing observations with replacements. For each bootstrap sample, we fitted the same logistic regression model as that for the original data and stored the coefficients. In total, 1000 bootstrap samples were utilized and empirical confidence intervals for regression coefficients were inferred accordingly. Using the bootstrapping function in SPSS, we estimated the "bias" which is the difference between the regression coefficients of the single model and the mean of the bootstrap samples.

**References for supplement**

1. Arshad SH, Holloway JW, Karmaus W, et al. Cohort Profile: The Isle Of Wight Whole Population Birth Cohort (IOWBC). Int J Epidemiol 2018;47(4):1043-1044i. (In eng). DOI: 10.1093/ije/dyy023.

2. Bousquet J, Heinzerling L, Bachert C, et al. Practical guide to skin prick tests in allergy to aeroallergens. Allergy 2012;67(1):18-24. (In eng). DOI: 10.1111/j.1398-9995.2011.02728.x.

3. Custovic A, Simpson BM, Murray CS, Lowe L, Woodcock A. The national asthma campaign Manchester asthma and allergy study. Pediatric Allergy and Immunology 2002;13(s15):32-37.

4. Boyd A, Golding J, Macleod J, et al. Cohort Profile: the 'children of the 90s'--the index offspring of the Avon Longitudinal Study of Parents and Children. Int J Epidemiol 2013;42(1):111-27. (In eng). DOI: 10.1093/ije/dys064.

5. Fraser A, Macdonald-Wallis C, Tilling K, et al. Cohort Profile: The Avon Longitudinal Study of Parents and Children: ALSPAC mothers cohort. International Journal of Epidemiology 2012;42(1):97-110. DOI: 10.1093/ije/dys066.

6. Arshad SH, Hodgekiss C, Holloway JW, et al. Association of asthma and smoking with lung function impairment in adolescence and early adulthood: the Isle of Wight Birth Cohort Study. Eur Respir J 2020;55(3) (In eng). DOI: 10.1183/13993003.00477-2019.

7. Miller MR, Hankinson J, Brusasco V, et al. Standardisation of spirometry. Eur Respir J 2005;26(2):319-38. (In eng). DOI: 10.1183/09031936.05.00034805.

8. Quanjer PH, Stanojevic S, Cole TJ, et al. Multi-ethnic reference values for spirometry for the 3-95-yr age range: the global lung function 2012 equations. Eur Respir J 2012;40(6):1324-43. (In eng). DOI: 10.1183/09031936.00080312.

**Table S1. Comparable definitions used for factors and outcomes and their prevalence in IOWBC, MAAS and ALSPAC cohorts**

| **Variables** | **IOWBC** | **N (%)** | **MAAS** | **N (%)** | **ALSPAC** | **N (%)** |
| --- | --- | --- | --- | --- | --- | --- |
| Asthma | Physician diagnosed asthma ever and either current wheeze or currently on asthma medication at ages 18 (and 26) years | 97(11.1%)/877 | (i) Have you ever had asthma and was this confirmed by a doctor and on asthma medication” at ages 18- years. | 115 (24.1%)/477 | Child had asthma in the past 12 months at ages 17-years | 304 (7.7%)/3933 |
| Recurrent wheeze | ≥3 Three or more separate episodes of wheeze occurring in the past 12 months at 4-years | 182 (15.0%)/1214 | Wheeze apart from cold at age 5-years | 107 (45%)/238 | Child had periods when there was wheezing/ whistling as they breathed (since age 3 years) at 5-years | 1515 (16.0%)/9470 |
| Skin prick test | Positive SPT to any allergens” at age 4- years | 171 (17.5%)/978 | Positive SPT to any allergens” at age 5- years | 293 (30.5%)/961 | Reaction to positive spt at 5-years | 1093 (20.5%)/5320 |
| Recurrent chest infections at 2 years | More than one chest infection reported in the past 12 months at 1- or 2-years. | 101 (7.4%)/1359 | - |  | - |  |
| Eczema | “Itchy rash present for more than 6 weeks with characteristic morphology and distribution” at age 4- years | 147 (12.1%)/1214 | “eczema” at age 5- years | 344 (32.2%)/1067 | eczema in the past 12 months” at 3.5-years. | 813 (14.8%)/5498 |
| Maternal Rhinitis | Yes to “hay fever/rhinitis” at recruitment | 305 (20.1%)/1517 | Maternal Rhinitis | 305 (26.3%)/1158 | Yes to “maternal hay fever” collected at recruitment | 3446 (30.4%)/11322 |

**Table S2. Association of candidate risk factors at first 4 years of life with asthma at 18 and 26-years old via Chi-square test.**

| **Risk Factors** | **Year 18** | | | | | | | | **Year 26** | | | | | | | |
| --- | --- | --- | --- | --- | --- | --- | --- | --- | --- | --- | --- | --- | --- | --- | --- | --- |
|  | **Asthmatic Participants** | | | **Non Asthmatic Participants** | | | **Crude OR (95% CI)** | **P-value** | **Asthmatic**  **Participants** | | | **Non-Asthmatic**  **Participants** | | | **Crude OR (95% CI)** | **P-value** |
| Sex [Female] | 51.9% (190/366) | | | 49.7% (470/946) | | | 1.09 (0.86-1.39) | 0.50 | 60.6% (97/160) | | | 53.2% (463/870) | | | 1.35 (0.96-1.91) | 0.09 |
| Socioeconomic factors | low | medium | high | low | medium | high | 0.84  (0.52-1.34) | 0.59 | low | medium | high | low | medium | high | 0.70  (0.36-1.40) | 0.60 |
|  | 13.4%  (45/337) | 97.2%  (267/337) | 7.4%  (25/337) | 14.7%  (132/895) | 76.5%  (685/895) | 8.7%  (78/895) |  |  | 14.0%  (20/143) | 79.0%  (113/143) | 7.0%  (10/143) | 14.2%  (117/822) | 67.2%  (662/822) | 9.6%  (79/822) |  |  |
| Mode of delivery (C-section) | 7.1 (8/113) | | | 8.5% (22/260) | | | 1.20 (0.52-2.77 | 0.68 | 4/42 (9.5%) | | | 17/243 (7.0%) | | | 0.73 (0.24-2.30) | 0.75 |
| Maternal asthma | 13.5% (49/363) | | | 9.4% (88/940) | | | 1.51 (1.04-2.19) | 0.03 | 17.0% (27/159) | | | 9.8% (85/863) | | | 1.87 (1.17-3.00) | 0.02 |
| Paternal asthma | 12.7% (46/362) | | | 8.8% (82/933) | | | 1.51 (1.03-2.22) | 0.04 | 12.6% (20/159) | | | 9.0% (77/857) | | | 1.46 (0.86-2.46) | 0.19 |
| Maternal eczema | 14.3% (52/363) | | | 11.6% (109/938) | | | 1.27 (0.89-1.81) | 0.19 | 15.1% (24/159) | | | 11.7% (101/861) | | | 1.34 (0.83-2.16) | 0.24 |
| Paternal eczema | 7.7% (28/362) | | | 6.7% (62/931) | | | 1.18 (0.74-1.87) | 0.54 | 5.0% (8/159) | | | 7.6% (65/855) | | | 0.64 (0.30-1.37) | 0.32 |
| Maternal Rhinitis | 23.7% (86/363) | | | 18.7% (176/940) | | | 1.35 (1.01-1.81) | 0.05 | 27.0% (43/159) | | | 19.2% (166/863) | | | 1.56 (1.06-2.30) | 0.03 |
| Paternal Allergic Rhinitis | 15.2% (55/362) | | | 14.4% (134/933) | | | 1.07 (0.76-1.50) | 0.73 | 12.6% (20/159) | | | 15.1% (129/856) | | | 0.81 (0.49-1.34) | 0.47 |
| Maternal food allergy | 5.2% (19/363) | | | 4.7% (44/940) | | | 1.12 (0.65-1.95) | 0.67 | 4.4% (7/159) | | | 5.0% (43/864) | | | 0.88 (0.39-1.99) | 1.00 |
| Paternal food allergy | 3.9% (14/362) | | | 2.0% (19/932) | | | 1.93 (0.96-3.90) | 0.08 | 4.4% (7/159) | | | 2.3% (20/856) | | | 1.93 (0.80-4.63) | 0.17 |
| Maternal smoking (year 0) | 25.1% (91/363) | | | 23.4% (219/934) | | | 1.09 (0.82-1.45) | 0.56 | 20.8% (33/159) | | | 23.3% (200/858) | | | 0.86 (0.57-1.30) | 0.54 |
| Paternal smoke (year 0) | 42.8% (155/362) | | | 37.3% (348/933) | | | 1.26 (0.98-1.61) | 0.08 | 40.9% (65/159) | | | 37.1% (318/856) | | | 1.17 (0.83-1.65) | 0.37 |
| Pets at home (year 0) | 57.9% (210/363) | | | 56.8% (533/939) | | | 1.05 (0.82-1.34) | 0.76 | 56.0% (89/159) | | | 56.6% (487/861) | | | 0.98 (0.69-1.37) | 0.93 |
| Recurrent chest infections (year 1) | 8.8% (28/317) | | | 6.7% (59/877) | | | 1.34 (0.84-2.15) | 0.26 | 9.6% (14/146) | | | 6.5% (52/802) | | | 1.53 (0.82-2.84) | 0.21 |
| Recurrent wheeze (year 1) | 5.4% (17/317) | | | 2.5% (22/878) | | | 2.20 (1.16-4.21) | 0.02 | 7.6% (11/145) | | | 2.6% (21/803) | | | 3.06 (1.44-6.49) | 0.01 |
| Eczema (year 1) | 18.5% (59/319) | | | 11.7% (103/880) | | | 1.71 (1.21-2.43) | <0.01 | 19.7% (29/147) | | | 12.1% (97/802) | | | 1.79 (1.13-2.82) | 0.02 |
| Recurrent nasal congestion (Year 1) | 19.7% (63/319) | | | 13.8% (122/883) | | | 1.54 (1.10-2.15) | 0.01 | 19.0% (28/147) | | | 15.2% (122/805) | | | 1.32 (0.84-2.08) | 0.27 |
| Food allergy (year 1) | 14.7% (47/319) | | | 10.5% (93/882) | | | 1.47 (1.01-2.14) | 0.05 | 16.3% (24/147) | | | 10.2% (82/804) | | | 1.72 (1.05-2.81) | 0.05 |
| Parental smoking at home (year 1) | 49.0% (153/312) | | | 39.4% (345/875) | | | 1.48 (1.14-1.92) | <0.01 | 45.1% (64/142) | | | 37.8% (301/797) | | | 1.35 (0.94-1.94) | 0.11 |
| Pets at home (year 1) | 52.9% (166/314) | | | 53.7% (472/879) | | | 0.97 (0.75-1.25) | 0.84 | 50.0% (73/146) | | | 54.1% (433/800) | | | 0.85 (0.60-1.21) | 0.37 |
| Recurrent wheeze (year 2) | 8.6% (24/279) | | | 3.5% (28/801) | | | 2.60 (1.48-4.56) | <0.01 | 9.3% (12/129) | | | 3.3% (24/729) | | | 3.01 (1.47-6.19) | 0.01 |
| Recurrent chest infections (Year 2) | 16.3% (46/283) | | | 11.9% (95/796) | | | 1.43 (0.98-2.10) | 0.08 | 18.8% (25/133) | | | 12.1% (88/730) | | | 1.69 (1.04-2.75) | 0.05 |
| Eczema (year 2) | 24.7% (71/288) | | | 19.9% (160/805) | | | 1.32 (0.96-1.81) | 0.09 | 24.6% (33/134) | | | 21.0% (155/737) | | | 1.23 (0.80-1.89) | 0.36 |
| Recurrent nasal congestion (Year 2) | 18.8% (54/288) | | | 14.6% (118/808) | | | 1.35 (0.95-1.92) | 0.11 | 14.2% (19/134) | | | 15.1% (112/741) | | | 0.93 (0.55-1.57) | 0.90 |
| Food allergy (year 2) | 14.8% (42/284) | | | 10.4% (84/808) | | | 1.50 (1.00-2.23) | 0.05 | 18.7% (25/134) | | | 10.0% (74/738) | | | 2.06 (1.25-3.38) | 0.01 |
| Smoking at home (year 2) | 45.5% (130/286) | | | 40.1% (319/796) | | | 1.25 (0.95-1.64) | 0.12 | 42.5% (57/134) | | | 38.7% (283/731) | | | 1.17 (0.81-1.70) | 0.44 |
| Pets at home (year 2) | 56.0% (159/284) | | | 55.5% (445/802) | | | 1.02 (0.78-1.34) | 0.89 | 53.0% (71/134) | | | 55.1% (402/730) | | | 0.92 (0.64-1.33) | 0.71 |
| Recurrent wheeze (year 4) | 30.5% (90/295) | | | 9.8% (80/814) | | | 4.03 (2.87-5.65) | <0.01 | 39.5% (49/124) | | | 10.8% (80/744) | | | 5.42 (3.53-8.32) | <0.01 |
| Eczema (year 4) | 17.6% (52/296) | | | 10.1% (82/814) | | | 1.90 (1.31-2.77) | <0.01 | 21.6% (27/125) | | | 10.8% (80/743) | | | 2.28 (1.41-3.71) | <0.01 |
| Recurrent nasal congestion (year 4) | 10.5% (31/296) | | | 3.6% (29/813) | | | 3.16 (1.87-5.35) | <0.01 | 8.8% (11/125) | | | 5.2% (39/743) | | | 1.74 (0.87-3.50) | 0.14 |
| Food allergy (year 4) | 5.4% (16/295) | | | 2.2% (18/814) | | | 2.54 (1.28-5.04) | <0.01 | 5.6% (7/126) | | | 2.4% (18/742) | | | 2.37 (0.97-5.79) | 0.08 |
| Positive SPT (year 4) | 33.8% (81/240) | | | 11.9% (79/665) | | | 3.78 (2.65-5.39) | <0.01 | 38.8% (40/103) | | | 13.6% (84/619) | | | 4.04 (2.56-6.39) | <0.01 |

Notes: Risk factors are the factors at childhood, which will have individual risk of developing asthma at adolescent and adulthood. Before preparing the final risk calculation model, to identify the significant factors among various available factors, Chi-square tests were applied. Only the significant factors with P-value less than 0.05 (Tables S2A and S2B) were included in the multivariable logistic regression model to identify the risk factors used to build ASPIRE-1 and ASPIRE-2.

**Table S2A. Statistically significant risk factors during the first 4 years of life associated with asthma at 18 years at univariate analysis. These factors were further included in the multivariable logistic regression model for risk score calculations.**

| **Risk Factors** | **Year 18** | | | |
| --- | --- | --- | --- | --- |
|  | **Asthmatic Participants** | **Non Asthmatic Participants** | **Crude OR (95% CI)** | **P-value** |
| Maternal asthma | 13.5% (49/363) | 9.4% (88/940) | 1.51 (1.04-2.19) | 0.03 |
| Paternal asthma | 12.7% (46/362) | 8.8% (82/933) | 1.51 (1.03-2.22) | 0.04 |
| Recurrent wheeze (year 1) | 5.4% (17/317) | 2.5% (22/878) | 2.20 (1.16-4.21) | 0.03 |
| Eczema (year 1) | 18.5% (59/319) | 11.7% (103/880) | 1.71 (1.21-2.43) | <0.01 |
| Recurrent nasal congestion (Year 1) | 19.7% (63/319) | 13.8% (122/883) | 1.54 (1.10-2.15) | 0.01 |
| Parental smoking at home (year 1) | 49.0% (153/312) | 39.4% (345/875) | 1.48 (1.14-1.92) | <0.01 |
| Recurrent wheeze (year 2) | 8.6% (24/279) | 3.5% (28/801) | 2.60 (1.48-4.56) | <0.01 |
| Recurrent wheeze (year 4) | 30.5% (90/295) | 9.8% (80/814) | 4.03 (2.87-5.65) | <0.01 |
| Eczema (year 4) | 17.6% (52/296) | 10.1% (82/814) | 1.90 (1.31-2.77) | <0.01 |
| Recurrent nasal congestion (year 4) | 10.5% (31/296) | 3.6% (29/813) | 3.16 (1.87-5.35) | <0.01 |
| Food allergy (year 4) | 5.4% (16/295) | 2.2% (18/814) | 2.54 (1.28-5.04) | 0.01 |
| Positive SPT (year 4) | 33.8% (81/240) | 11.9% (79/665) | 3.78 (2.65-5.39) | <0.01 |

Note: Statistical methods to select these variables were univariate binary logistic regression models

**Table S2B. Statistically significant risk factors during the first 4 years of life associated with asthma at 26 years at univariate analysis. These factors were further included in the multivariable logistic regression model for risk score calculations.**

| **Risk Factors** | **Year 26** | | | |
| --- | --- | --- | --- | --- |
|  | **Asthmatic**  **Participants** | **Non-Asthmatic**  **Participants** | **Crude OR (95% CI)** | **P-value** |
| Maternal asthma | 17.0% (27/159) | 9.8% (85/863) | 1.87 (1.17-3.00) | 0.01 |
| Maternal Rhinitis | 27.0% (43/159) | 19.2% (166/863) | 1.56 (1.06-2.30) | 0.03 |
| Recurrent wheeze (year 1) | 7.6% (11/145) | 2.6% (21/803) | 3.06 (1.44-6.49) | <0.01 |
| Eczema (year 1) | 19.7% (29/147) | 12.1% (97/802) | 1.79 (1.13-2.82) | 0.02 |
| Food allergy (year 1) | 16.3% (24/147) | 10.2% (82/804) | 1.72 (1.05-2.81) | 0.04 |
| Recurrent wheeze (year 2) | 9.3% (12/129) | 3.3% (24/729) | 3.01 (1.47-6.19) | <0.01 |
| Food allergy (year 2) | 18.7% (25/134) | 10.0% (74/738) | 2.06 (1.25-3.38) | <0.01 |
| Recurrent wheeze (year 4) | 39.5% (49/124) | 10.8% (80/744) | 5.42 (3.53-8.32) | <0.01 |
| Eczema (year 4) | 21.6% (27/125) | 10.8% (80/743) | 2.28 (1.41-3.71) | <0.01 |
| Positive SPT (year 4) | 38.8% (40/103) | 13.6% (84/619) | 4.04 (2.56-6.39) | <0.01 |

Note: Statistical methods to select these variables were univariate binary logistic regression models

**Table S3. Risk factors at first 4-years of life associated with persistent asthma included in the analysis.**

| **Risk Factors** | **Persistent Asthma year 10 and 18** | | | | | | | | **Persistent Asthma year 10, 18 and 26** | | | | | | | |
| --- | --- | --- | --- | --- | --- | --- | --- | --- | --- | --- | --- | --- | --- | --- | --- | --- |
|  | **Persistent Asthma** | | | **No Asthma** | | | **Crude OR (95% CI)** | **P-value** | **Persistent Asthma** | | | **Non-Asthmatic Participants** | | | **Crude OR (95% CI)** | **P-value** |
| Sex [Female] | 43.7% (94/215) | | | 50.3% (374/743) | | | 0.8 (0.6-1.0) | 0.09 | 53.6% (52/97) | | | 50.5% (394/780) | | | 1.13 (0.74-1.73) | 0.57 |
| Socioeconomic status | low | medium | high | low | medium | high | 0.79 (0.45-1.39) | 0.55 | low | medium | high | low | medium | high | 0.89 (0.42-1.92) | 0.84 |
|  | 12.1%  (25/206) | 80.1%  (165/206) | 7.8%  (16/206) | 13.8%  (99/716) | 76.5%  (548/716) | 9.6%  (69/716) |  |  | 12.9%  (12/93) | 78.5%  (73/93) | 8.6%  (8/93) | 14.7%  (108/734) | 75.7%  (556/737) | 9.5%  (70/737) |  |  |
| Mode of delivery (C-section) | 6.6% (5/76) | | | 7.8 (16/205) | | | 1.20-(0.42-3.40) | 0.73 | 6.5% (2/31) | | | 7.9 (17/216) | | | 1.24 (0.27-5.64) | 0.78 |
| Maternal asthma | 15.0% (32/213) | | | 8.9% (66/739) | | | 1.8 (1.1-2.8) | 0.01 | 13.5% (13/96) | | | 9.0% (70/776) | | | 1.58 (0.84-2.98) | 0.15 |
| Paternal asthma | 15.6% (33/212) | | | 8.2% (60/733) | | | 2.1 (1.3-3.3) | <0.01 | 13.5% (13/96) | | | 8.2% (63/770) | | | 1.76 (0.93-3.33) | 0.08 |
| Maternal eczema | 14.1% (30/213) | | | 10.2% (75/738) | | | 1.4 (0.9-2.3) | 0.11 | 14.6% (14/96) | | | 11.0% (85/775) | | | 1.39 (0.75-2.55) | 0.29 |
| Paternal eczema | 8.5% (18/212) | | | 6.7% (49/731) | | | 1.3 (0.7-2.3) | 0.37 | 5.2% (5/96) | | | 6.5% (50/768) | | | 0.79 (0.31-2.03) | 0.62 |
| Maternal rhinitis | 26.3% (56/213) | | | 18.5% (137/739) | | | 1.6 (1.1-2.2) | 0.02 | 30.2% (29/96) | | | 18.8% (146/776) | | | 1.87 (1.17-2.99) | <0.01 |
| Paternal allergic rhinitis | 17.5% (37/212) | | | 14.6% (107/732) | | | 1.2 (0.8-1.9) | 0.33 | 13.5% (13/96) | | | 14.8% (114/769) | | | 0.90 (0.49-1.67) | 0.74 |
| Maternal food allergy | 4.7% (10/213) | | | 5.3% (39/739) | | | 0.9 (0.4-1.8) | 0.86 | 2.1% (2/96) | | | 4.9% (38/776) | | | 0.41 (0.10-1.74) | 0.21 |
| Paternal food allergy | 4.2% (9/212) | | | 2.2% (16/731) | | | 2.0 (0.9-4.5) | 0.14 | 5.2% (5/96) | | | 2.1% (16/768) | | | 2.58 (0.92-7.22) | 0.06 |
| Maternal smoking (year 0) | 22.1% (47/213) | | | 21.6% (158/733) | | | 1.0 (0.7-1.5) | 0.93 | 15.6% (15/96) | | | 22.9% (176/770) | | | 0.63 (0.35-1.11) | 0.11 |
| Paternal smoking (year 0) | 41.0% (87/212) | | | 36.0% (264/733) | | | 1.2 (0.9-1.7) | 0.20 | 39.6% (38/96) | | | 36.6% (282/770) | | | 1.13 (0.73-1.75) | 0.57 |
| Pets at home (year 0) | 56.3% (120/213) | | | 57.4% (424/739) | | | 1.0 (0.7-1.3) | 0.81 | 58.3% (56/96) | | | 56.9% (441/775) | | | 1.06 (0.69-1.63) | 0.789 |
| Recurrent chest infections (year 1) | 12.0% (23/191) | | | 5.4% (37/690) | | | 2.4 (1.4-4.2) | <0.01 | 10.3% (9/87) | | | 5.8% (42/722) | | | 1.87 (0.88-3.98) | 0.10 |
| Recurrent wheeze (year 1) | 7.3% (14/192) | | | 2.0% (14/691) | | | 3.8 (1.8-8.1) | <0.01 | 8.0% (7/87) | | | 1.9% (14/723) | | | 4.43 (1.74-11.30) | <0.01 |
| Eczema (year 1) | 23.8% (46/193) | | | 10.7% (74/691) | | | 2.6 (1.7-3.9) | <0.01 | 25.0% (22/88) | | | 10.4% (75/723) | | | 2.88 (1.68-4.93) | <0.01 |
| Recurrent nasal congestion (Year 1) | 19.2% (37/193) | | | 13.0% (90/694) | | | 1.6 (1.0-2.4) | 0.04 | 19.3% (17/88) | | | 13.1% (95/726) | | | 1.59 (0.90-2.82) | 0.11 |
| Food allergy (year 1) | 16.1% (31/193) | | | 9.8% (68/693) | | | 1.8 (1.1-2.8) | 0.02 | 15.9% (14/88) | | | 9.2% (67/725) | | | 1.86 (1.00-3.47) | 0.05 |
| Parental smoking at home (year 1) | 46.3% (87/188) | | | 37.4% (258/690) | | | 1.4 (1.0-2.0) | 0.03 | 39.3% (33/84) | | | 38.0% (274/722) | | | 1.06 (0.67-1.68) | 0.81 |
| Pets at home (year 1) | 54.5% (103/189) | | | 54.6% (377/691) | | | 1.0 (0.7-1.4) | 1.00 | 53.4% (47/88) | | | 53.9% (390/724) | | | 0.98 (0.63-1.53) | 0.94 |
| Recurrent wheeze (year 2) | 11.0% (18/164) | | | 1.9% (12/629) | | | 6.3 (3.0-13.5) | <0.01 | 11.8% (9/76) | | | 1.8% (12/657) | | | 7.22 (2.94-17.76) | <0.01 |
| Recurrent chest infections (Year 2) | 22.2% (38/171) | | | 8.9% (56/626) | | | 2.9 (1.8-4.6) | <0.01 | 21.0% (17/81) | | | 8.9% (58/655) | | | 2.73 (1.50-4.98) | <0.01 |
| Eczema (year 2) | 30.6% (53/173) | | | 19.2% (121/630) | | | 1.9 (1.3-2.7) | <0.01 | 30.9% (25/81) | | | 19.0% (125/659) | | | 1.91 (1.15-3.18) | 0.02 |
| Recurrent nasal congestion (Year 2) | 22.5% (39/173) | | | 12.8% (81/633) | | | 2.0 (1.3-3.0) | <0.01 | 16.0% (13/81) | | | 13.0% (86/662) | | | 1.28 (0.68-2.42) | 0.45 |
| Food allergy (year 2) | 18.9% (32/169) | | | 10.4% (66/634) | | | 2.0 (1.3-3.2) | <0.01 | 24.7% (20/81) | | | 10.0% (66/663) | | | 2.97 (1.68-5.22) | <0.01 |
| Smoking at home (year 2) | 43.0% (74/172) | | | 37.6% (234/622) | | | 1.3 (0.9-1.8) | 0.22 | 38.3% (31/81) | | | 38.3% (249/650) | | | 1.00 (0.62-1.61) | 0.10 |
| Pets at home (year 2) | 55.0% (94/171) | | | 56.0% (351/627) | | | 1.0 (0.7-1.3) | 0.86 | 54.3% (44/81) | | | 55.3% (363/656) | | | 0.96 (0.60-1.53) | 0.86 |
| Recurrent wheeze (year 4) | 44.5% (81/182) | | | 4.8% (31/651) | | | 16.0 (10.1-25.5) | <0.01 | 46.3% (38/82) | | | 4.7% (31/666) | | | 17.69 (10.06-31.10) | <0.01 |
| Eczema (year 4) | 23.0% (42/183) | | | 8.5% (55/650) | | | 3.2 (2.1-5.0) | <0.01 | 26.5% (22/83) | | | 8.6% (57/665) | | | 3.85 (2.20-6.72) | <0.01 |
| Recurrent nasal congestion (year 4) | 12.6% (23/183) | | | 2.5% (16/651) | | | 5.7 (2.9-11.1) | <0.01 | 12.0% (10/83) | | | 2.3% (15/666) | | | 5.95 (2.58-13.72) | <0.01 |
| Food allergy (year 4) | 7.7% (14/183) | | | 1.7% (11/651) | | | 4.8 (2.1-10.8) | <0.01 | 7.2% (6/83) | | | 1.7% (11/665) | | | 4.63 (1.67-12.88) | <0.01 |
| Positive SPT (year 4) | 43.3% (65/150) | | | 9.9% (52/527) | | | 7.0 (4.5-10.8) | <0.01 | 48.6% (35/72) | | | 9.8% (53/540) | | | 8.69 (5.05-14.95) | <0.01 |

Notes: Risk factors are the factors at childhood, which will have individual risk of developing asthma at adolescent and adulthood. Before preparing the final risk calculation model, to identify the significant factors among various available factors, chi-square tests applied. Only the significant factors with P-value less than 0.05 (Tables S3A and S3B) were included in the multivariable logistic regression model to identify the risk factors used to build ASPIRE 3 and ASPIRE 4.

**Table S3A. Statistically significant risk factors during the first 4 years of life associated with persistent asthma at 10- to 18 years (PA-18) at univariate analysis. These factors were further included in the multivariable logistic regression model for risk score calculations.**

| **Risk Factors** | **Persistent Asthma year 10 and 18** | | | |
| --- | --- | --- | --- | --- |
|  | **Persistent Asthma** | **No Asthma** | **Crude OR (95% CI)** | **P-value** |
| Maternal asthma | 15.0% (32/213) | 8.9% (66/739) | 1.8 (1.1-2.8) | 0.01 |
| Paternal asthma | 15.6% (33/212) | 8.2% (60/733) | 2.1 (1.3-3.3) | <0.01 |
| Maternal rhinitis | 26.3% (56/213) | 18.5% (137/739) | 1.6 (1.1-2.2) | 0.02 |
| Recurrent chest infections (year 1) | 12.0% (23/191) | 5.4% (37/690) | 2.4 (1.4-4.2) | <0.01 |
| Recurrent wheeze (year 1) | 7.3% (14/192) | 2.0% (14/691) | 3.8 (1.8-8.1) | <0.01 |
| Eczema (year 1) | 23.8% (46/193) | 10.7% (74/691) | 2.6 (1.7-3.9) | <0.01 |
| Recurrent nasal congestion (Year 1) | 19.2% (37/193) | 13.0% (90/694) | 1.6 (1.0-2.4) | 0.04 |
| Food allergy (year 1) | 16.1% (31/193) | 9.8% (68/693) | 1.8 (1.1-2.8) | 0.02 |
| Parental smoking at home (year 1) | 46.3% (87/188) | 37.4% (258/690) | 1.4 (1.0-2.0) | 0.03 |
| Recurrent wheeze (year 2) | 11.0% (18/164) | 1.9% (12/629) | 6.3 (3.0-13.5) | <0.01 |
| Recurrent chest infections (Year 2) | 22.2% (38/171) | 8.9% (56/626) | 2.9 (1.8-4.6) | <0.01 |
| Eczema (year 2) | 30.6% (53/173) | 19.2% (121/630) | 1.9 (1.3-2.7) | <0.01 |
| Recurrent nasal congestion (Year 2) | 22.5% (39/173) | 12.8% (81/633) | 2.0 (1.3-3.0) | <0.01 |
| Food allergy (year 2) | 18.9% (32/169) | 10.4% (66/634) | 2.0 (1.3-3.2) | <0.01 |
| Recurrent wheeze (year 4) | 44.5% (81/182) | 4.8% (31/651) | 16.0 (10.1-25.5) | <0.01 |
| Eczema (year 4) | 23.0% (42/183) | 8.5% (55/650) | 3.2 (2.1-5.0) | <0.01 |
| Recurrent nasal congestion (year 4) | 12.6% (23/183) | 2.5% (16/651) | 5.7 (2.9-11.1) | <0.01 |
| Food allergy (year 4) | 7.7% (14/183) | 1.7% (11/651) | 4.8 (2.1-10.8) | <0.01 |
| Positive SPT (year 4) | 43.3% (65/150) | 9.9% (52/527) | 7.0 (4.5-10.8) | <0.01 |

Note: Statistical methods to select these variables were univariate binary logistic regression models

**Table S3B. Statistically significant risk factors during the first 4 years of life associated with persistent asthma at 10 to 26 years (PA-26) at univariate analysis. These factors were further included in the multivariable logistic regression model for risk score calculations.**

| **Risk Factors** | **Persistent Asthma year 10, 18 and 26** | | | |
| --- | --- | --- | --- | --- |
|  | **Persistent Asthma** | **Non-Asthmatic Participants** | **Crude OR (95% CI)** | **P-value** |
| Maternal rhinitis | 30.2% (29/96) | 18.8% (146/776) | 1.87 (1.17-2.99) | <0.01 |
| Recurrent wheeze (year 1) | 8.0% (7/87) | 1.9% (14/723) | 4.43 (1.74-11.30) | <0.01 |
| Eczema (year 1) | 25.0% (22/88) | 10.4% (75/723) | 2.88 (1.68-4.93) | <0.01 |
| Food Allergy (year 1) | 15.9% (14/88) | 9.2% (67/725) | 1.86 (1.00-3.47) | <0.05 |
| Recurrent wheeze (year 2) | 11.8% (9/76) | 1.8% (12/657) | 7.22 (2.94-17.76) | <0.01 |
| Recurrent chest infections (Year 2) | 21.0% (17/81) | 8.9% (58/655) | 2.73 (1.50-4.98) | <0.01 |
| Eczema (year 2) | 30.9% (25/81) | 19.0% (125/659) | 1.91 (1.15-3.18) | 0.01 |
| Food allergy (year 2) | 24.7% (20/81) | 10.0% (66/663) | 2.97 (1.68-5.22) | <0.01 |
| Recurrent wheeze (year 4) | 46.3% (38/82) | 4.7% (31/666) | 17.69 (10.06-31.10) | <0.01 |
| Eczema (year 4) | 26.5% (22/83) | 8.6% (57/665) | 3.85 (2.20-6.72) | <0.01 |
| Recurrent nasal congestion (year 4) | 12.0% (10/83) | 2.3% (15/666) | 5.95 (2.58-13.72) | <0.01 |
| Food allergy (year 4) | 7.2% (6/83) | 1.7% (11/665) | 4.63 (1.67-12.88) | <0.01 |
| Positive (SPT year 4) | 48.6% (35/72) | 9.8% (53/540) | 8.69 (5.05-14.95) | <0.01 |

Note: Statistical methods to select these variables were univariate binary logistic regression models

| Variable | All (N=1456) | Analysis population (N=1030) | Participants included in ASPIRE-1; to predict Asthma-18  (N=905) | Participants included in PARS; to predict Asthma-18  (N=1273) | Participants included in ASPIRE-2; to predict Asthma-26  (N=832) | Participants included in PARS; to predict Asthma-26  (N=998) | Participants included when using RW and +SPT; to predict Asthma-26  (N=677) |
| --- | --- | --- | --- | --- | --- | --- | --- |
| Male sex | 745 (51.2%) | 470 (45.6%) | 454 (50.2%) | 648 (50.9%) | 416 (50.0%) | 508 (50.9%) | 346 (51.1%) |
| Recurrent wheeze at 4 years | 182 (15.0%) | 129 (14.9%) | 147 (16.3%) | 182 (15.0%) | 134 (16.2%) | 150 (15.0%) | 91 (13.4%) |
| Positive SPT at year 4 | 171 (17.5%) | 124 (17.2%) | 158 (17.5%) | 171 (17.5%) | 146 (17.6%) | 174 (17.5%) | 117 (17.3%) |
| Maternal rhinitis | 305 (20.1%) | 209 (20.5%) | 189 (20.9%) | 291 (20.2%) | 174 (20.9%) | 202 (20.2%) | 137 (20.3%) |
| Eczema at 4 years | 147 (12.1%) | 107 (12.3%) | 114 (12.6%) | 147 (12.1%) | 104 (12.4%) | 121 (12.1%) | 79 (11.6%) |
| Recurrent chest infections at 2 years | 154 (12.7%) | 113 (13.1%) | 116 (12.8%) | 154 (12.7%) | 106 (12.7%) | 127 (12.7%) | 80 (11.8%) |

**Table S4. Characteristics of participants in the analysis data set for ASPIRE 1 and 2**

Notes: ASPIRE: Adult aSthma PredIctive Risk score; PARS: predictive asthma risk score; SPT: Skin Prick Test; Asthma-18: Asthma at age 18; Asthma-26: Asthma at age 26.The % were calculated as the sample size available for each variable divided by N in each column.

**Table S5. Characteristics of participants in the analysis data set for ASPIRE 3 and 4.**

| Variable | All (N=1456) | Analysis population (N=1030) | Participants included in ASPIRE-3; to predict PA-18  (N=677) | Participants included in PARS; to predict PA-18 (N=937) | Participants included when using RW and +spt; to predict PA-18 (N=677) | Participants included in ASPIRE-4; to predict PA-26 (N=557) | Participants included in PARS; to predict PA-26 (N=857) | Participants included when using RW and +SPT; to predict PA-26 (N=612) |
| --- | --- | --- | --- | --- | --- | --- | --- | --- |
| Male sex | 745 (51.2%) | 470 (45.6%) | 340 (50.2%) | 477 (50.9%) | 340 (50.2%) | 275 (49.4%) | 419 (48.9%) | 328 (53.9%) |
| Recurrent wheeze at 4 years | 182 (15.0%) | 129 (14.9%) | 110 (16.3%) | 140 (15.0%) | 110 (16.3%) | 85 (15.2%) | 169 (19.7%) | 127 (20.9%) |
| Positive SPT at 4 years | 171 (17.5%) | 124 (17.2%) | 118 (17.5%) | 164 (17.5%) | 118 (17.5%) | 99 (17.7%) | 171 (20.0%) | 114 (18.7%) |
| Maternal rhinitis | 305 (20.1%) | 209 (20.5%) | 141 (20.9%) | 190 (20.2%) | 141 (20.9%) | 113 (20.4%) | 199 (23.2%) | 166 (27.3%) |
| Eczema at 4 years | 147 (12.1%) | 107 (12.3%) | 85 (12.6%) | 113 (12.1%) | 85 (12.6%) | 69 (12.3%) | 129 (15.0%) | 118 (19.4%) |
| Recurrent chest infections at 2 years | 154 (12.7%) | 113 (13.1%) | 87 (12.8%) | 119 (12.7%) | 87 (12.8%) | 71 (12.8%) | 131 (15.2%) | 111 (18.2%) |

Notes: ASPIRE: Adult aSthma PredIctive Risk score; PARS: predictive asthma risk score; SPT: Skin Prick Test; PA-18: Persistent asthma up to age 18; PA-26: Persistent asthma up to age 26. The % were calculated as the sample size available for each variable divided by N in each column.

**Table S6. Association of atopic wheeze (RW and +SPT) at 4 years with lung function at 10, 18 and 26-years**

|  | **10-years** | | | |  | **18-years** | | | |  | **26-years** | | | |
| --- | --- | --- | --- | --- | --- | --- | --- | --- | --- | --- | --- | --- | --- | --- |
|  | Mean | | Difference | P value |  | Mean | | Difference | P value |  | Mean | | Difference | P value |
|  | No atopic wheeze  N=816 | Atopic wheeze (RW and +SPT)  N=53 |  |  |  | No atopic wheeze  N=710 | Atopic wheeze (RW and +SPT) N=38 |  |  |  | No atopic wheeze  N=462 | Atopic wheeze (RW and +SPT)  N-63 |  |  |
|  |  |  |  |  |  |  |  |  |  |  |  |  |  |  |
| **FEV_1_ % predicted** | 98.9 | 95.8 | 3.1 | 0.06 |  | 104.7 | 93.6 | 11.1 | <0.001 |  | 100.9 | 88.0 | 12.9 | <0.001 |
| **FVC % predicted** | 100.7 | 101.4 | -0.7 | 0.63 |  | 110.4 | 109.2 | 1.2 | 0.63 |  | 106.1 | 98.0 | 8.1 | 0.001 |
| **FEV1/FVC ratio** | 0.89 | 0.85 | 0.04 | <0.001 |  | 0.88 | 0.79 | 0.09 | <0.001 |  | 0.80 | 0.76 | 0.05 | 0.001 |
| **FEF_25-75_ % predicted** | 101.5 | 89.3 | 12.2 | 0.004 |  | 109.1 | 79.9 | 29.1 | <0.001 |  | 92.0 | 71.0 | 21.0 | <0.001 |

Notes: FEV_1_: Forced expiratory volume in one second. FVC: Forced vital capacity; FEF_25-75_: Forced expiratory flow 25 to 75%.

Statistical methods used were independent sample t-test.

**Table S7. Characteristics of participants in the analysis data set for ASPIRE 1 and 3 replicated in MAAS.**

| Variables | All (N=1163) | Participants included in ASPIRE-1 replicated in MAAS for prediction of Asthma-18 (N=548) | Participants included in ASPIRE-3 replicated in MAAS for replication of PA-18 (N=439) |
| --- | --- | --- | --- |
| Recurrent wheeze at 4-years | 107 (9.2%) | 61 (11.1%) | 47 (10.2%) |
| Positive SPT at 4-years | 293 (25.2%) | 166 (30.3%) | 136 (30.0%) |
| Maternal rhinitis | 305 (26.2%) | 156 (28.5%) | 129 (26.9%) |
| Eczema at 4-years | 344 (29.6%) | 176 (32.1%) | 151 (32.0%) |
| Recurrent chest infections at 2-years | 188 (16.2%) | 90 (16.4%) | 75 (15.4%) |

Notes: ASPIRE: Adult aSthma PredIctive Risk score; MAAS: Manchester Asthma and Allergy study; RW: Recurrent wheeze; SPT: Skin Prick Test; Asthma-18: Asthma at 18-years; PA-18: Persistent asthma at age 18-years.

**Table S8. Characteristics of participants in the analysis data set for ASPIRE 1 and 3 replicated in ALSPAC.**

| Variable | Analysis population (N=5028) | Participants included in ASPIRE 1 replicated in ALSPAC for prediction of Asthma-18  (N=4370) | Participants included in ASPIRE 3 replicated in ALSPAC for prediction of PA-18  (N=3642) |
| --- | --- | --- | --- |
| Recurrent wheeze at 4-years | 616 (12.3%) | 616 (14.1%) | 440 (12.1%) |
| Positive SPT at 4-years | 375 (7.5%) | 375 (8.6%) | 306 (8.4%) |
| Maternal rhinitis | 1150 (22.9%) | 1021 (23.4%) | 833 (22.8%) |
| Eczema at 4-years | 480 (9.5%) | 469 (10.7%) | 395 (10.8%) |

Notes: ASPIRE: Adult aSthma PredIctive Risk score; ALSPAC: The Avon Longitudinal Study of Parents and Children; SPT:

Skin Prick Test; Asthma-18: Asthma at 18-years; PA-18: Persistent asthma at age 18-years.
